# Australian public perspectives on genomic newborn screening: which conditions should be included?

**DOI:** 10.1186/s40246-024-00611-x

**Published:** 2024-05-08

**Authors:** Fiona Lynch, Stephanie Best, Clara Gaff, Lilian Downie, Alison D. Archibald, Christopher Gyngell, Ilias Goranitis, Riccarda Peters, Julian Savulescu, Sebastian Lunke, Zornitza Stark, Danya F. Vears

**Affiliations:** 1 Biomedical Ethics Research Group, Murdoch Children’s Research Institute, Parkville, VIC 3052 Australia; 2 Melbourne Law School, The University of Melbourne, Melbourne, VIC 3052 Australia; 3 Sir Peter MacCallum Cancer Centre Dept of Oncology, University of Melbourne, Melbourne, VIC 3052 Australia; 4 Australian Genomics, Melbourne, VIC 3052 Australia; 5 School of Health Sciences, University of Melbourne, Melbourne, VIC 3052 Australia; 6 Murdoch Children’s Research Institute, Parkville, VIC 3052 Australia; 7 Melbourne Genomics, Melbourne, VIC 3052 Australia; 8 Department of Paediatrics, Faculty of Medicine, Dentistry and Health Sciences, The University of Melbourne, Melbourne, VIC 3052 Australia; 9 Victorian Clinical Genetics Services, Murdoch Children’s Research Institute, Parkville, VIC 3052 Australia; 10 Economics of Genomics and Precision Medicine Unit, Centre for Health Policy, Melbourne School of Population and Global Health, The University of Melbourne, Melbourne, VIC 3052 Australia; 11 Centre for Biomedical Ethics, Yong Loo Lin School of Medicine, National University of Singapore, Singapore, 117597 Singapore; 12 Uehiro Chair of Practical Ethics, The Oxford Uehiro Centre for Practical Ethics, Oxford University, Oxford, OX1 1PT UK; 13 Department of Pathology, The University of Melbourne, Melbourne, VIC 3052 Australia; 14 Centre for Biomedical Ethics and Law, Department of Public Health and Primary Care, KU Leuven, Leuven, 3000 Belgium

**Keywords:** Newborn screening, Bioethics, Genomic sequencing, Qualitative, Public views

## Abstract

**Background:**

Implementing genomic sequencing into newborn screening programs allows for significant expansion in the number and scope of conditions detected. We sought to explore public preferences and perspectives on which conditions to include in genomic newborn screening (gNBS).

**Methods:**

We recruited English-speaking members of the Australian public over 18 years of age, using social media, and invited them to participate in online focus groups.

**Results:**

Seventy-five members of the public aged 23–72 participated in one of fifteen focus groups. Participants agreed that if prioritisation of conditions was necessary, childhood-onset conditions were more important to include than later-onset conditions. Despite the purpose of the focus groups being to elicit public preferences, participants wanted to defer to others, such as health professionals or those with a lived experience of each condition, to make decisions about which conditions to include. Many participants saw benefit in including conditions with no available treatment. Participants agreed that gNBS should be fully publicly funded.

**Conclusion:**

How many and which conditions are included in a gNBS program will be a complex decision requiring detailed assessment of benefits and costs alongside public and professional engagement. Our study provides support for implementing gNBS for treatable childhood-onset conditions.

**Supplementary Information:**

The online version contains supplementary material available at 10.1186/s40246-024-00611-x.

## Background

Standard newborn screening (stdNBS) programs have been operational for over 50 years, have high uptake, and are delivered at low cost. With the increasing availability and decreasing cost of genomic sequencing technologies, discussions about incorporating genomics into NBS have been pervasive in the literature. One of the main proposed advantages of genomic newborn screening (gNBS) is that it would allow for the significant expansion of the number of conditions able to be detected early in a child’s life [1]. 

Population-wide screening programs have traditionally been informed by the Wilson and Jungner criteria [2] for deciding which conditions to include. One criterion relates to the potential for some form of treatment to be implemented in response to early detection. Although over 700 rare conditions have some treatment available, less than 5% of these are included in NBS programs [3]. Genomic sequencing may help to alleviate this gap while also allowing for new conditions to be assimilated should treatment become available [1]. 

However, some have suggested that the Wilson and Jungner criteria are outdated in the genomics era, as they rely on decisions being made on a condition-by-condition basis [4]. In reality, the vast number of conditions that could be screened using genomic sequencing makes this case by case model impractical moving forward [5]. The evolving clinical landscape and growing interest and availability of genomic sequencing technologies has prompted reconsideration of the appropriateness of these criteria in the modern age since these technologies were introduced [4]. As such, revised principles have been proposed [4, 6, 7]. 

When considering the inclusion of genomic sequencing in NBS programs, it is tempting to adopt a ‘bigger is better’ approach to identify as many conditions as possible as early as possible. However, the more conditions screened, the more complex the technical, psychosocial and ethical aspects become [5]. Therefore it is critical that decisions about which conditions to include in gNBS should consider both expert and public opinion [5]. We aimed to explore the Australian public’s preferences and perspectives on increasing the number of conditions screened in NBS programs using genomic sequencing.

## Methods

### Recruitment

The methods of this paper have been published in detail elsewhere [8]. In brief, we recruited English-speaking members of the Australian public over 18 years of age via social media posts on Facebook. Those who were interested in participating were directed to a website to register their interest and then contacted to confirm eligibility and maximise participant heterogeneity (e.g., age; gender; location; parent status; and country of birth and language spoken at home (as measures of cultural and linguistic diversity). Individuals signed an online consent form, provided their availabilities to schedule the focus groups, and were asked to watch a three-minute video [9] to provide them with background information about genomics and current newborn screening programs in Australia. Participants were allocated to focus groups based purely on their availability. Participants were sent a AUD$75 (-USD$50) voucher at focus group completion as remuneration for their time.

### Data collection and analysis

Focus groups, conducted and recorded via Zoom, explored participants’ preferences and values regarding key gNBS characteristics and preferences for gNBS service delivery. Both DV and FL are skilled qualitative researchers with experience in focus group methodology and training in genetic counselling. None of the participants were known to the researchers. The focus group guide is included in the supplementary materials. Participants received very little prompting aside from the questions shown and examples of conditions relating to the factors were not provided by the researchers. Interview transcripts were analysed using inductive content analysis, whereby content categories are generated from the data, rather than predetermined [10]. Coding continued iteratively until all data relevant to the research question had been coded into categories and subcategories. All transcripts were coded by FL; DV checked the coding to ensure rigour. The coding was discussed by DV and FL who, together used the categories and subcategories to generate the overall findings. Data analysis was managed using NVivo (released March 2023) [11]. 

## Results

### Participant demographics

Seventy-five members of the Australian public aged 23–72 participated in one of fifteen focus groups (range 2–8 per group). Participant characteristics are summarised in Table 1. Thirteen participants self-reported they were health professionals. Seventeen participants disclosed they had a child with a genetic condition. Eleven participants were on parental leave at the time of the focus group.

Below we report data from the following categories resulting from inductive content analysis: (1) Deciding which conditions to include in gNBS; (2) Criteria for which conditions to include, and (3) Cost and funding of gNBS. Representative quotes are used to illustrate our findings. An ellipsis (…) reflects where a significant portion of speech has been removed, and square brackets represent where a word has been replaced for clarity or to protect participant anonymity. Quotes are deidentified to protect participant anonymity; codes are used to identify participants based on their focus group number (e.g., FG1 P1 refers to focus group 1, participant 1).

**Table 1:** Participant characteristics

	n (%)
Age	
Mean	42
Range	23–72
State/territory	
Victoria	40 (53%)
New South Wales	15 (20%)
Queensland	5 (7%)
Australian Capital Territory	5 (7%)
South Australia	4 (5%)
Western Australia	4 (5%)
Tasmania	2 (3%)
Metro/rural	
Metro	61 (81%)
Regional/rural	14 (19%)
Children	
Y	62 (83%)
N	13 (17%)
Country of birth	
Australia	61 (81%)
Other	14 (19%)
Language spoken at home	
English only	60 (80%)
Language other than English	15 (20%)
Gender	
Female	66 (88%)
Male	9 (12%)
TOTAL	75

### Deciding which conditions to include

Participants discussed how to decide which conditions are included in gNBS. Illustrative quotes are shown in Table 2. Some participants felt that every possible condition should be included in gNBS (Table 2, Quote 1). This often included both treatable and untreatable conditions (Table 2, Quote 2).

Many participants assumed that conditions would need to be prioritised for inclusion, and therefore discussed exactly how to make these decisions. Despite the purpose of the focus groups being to elicit these public preferences, participants wanted to defer to others to make these decisions, such as health professionals or those with a lived experience of each condition (Table 2, Quotes 3 and 4).

Although some participants thought it was not appropriate to allow parental choice, others stated that parents should be given the choice about which conditions they wanted their newborn screened for (Table 2, Quote 5). However, some recognised that presenting parents with a long list of conditions could be overwhelming, and so suggested ‘grouping’ conditions to make the choice easier (Table 2, Quotes 6 and 7).

**Table 2:** Deciding which conditions to include

	Illustrative quote
Every condition should be included	*Quote 1: “I actually don’t think there is a genetic presentation unless it’s without manifestation that doesn’t deserve some level of attention.” [FG1 P8]* *Quote 2: “…I would just want as many conditions as possible to be tested…treatable or not treatable, severe or not severe, anything that can be included that’s possible I would really hope for…I would just hope that as much as possible could be included.” [FG2 P4]*
Who should decide which conditions to include	*Quote 3: “I don’t have any lived experience directly of any conditions, and I think the opinions of people who live, maybe, with these conditions might be, I think that their opinions may in some way be more valuable than mine…so maybe getting more opinions than just, like the people who have had more of a lived experience of this would be really, really helpful, I think. I want to defer more to them…” [FG3 P2]* *Quote 4: “…it’s really more up to the medical profession, yeah, I don’t have that medical background so they would know best what to screen for…” [FG6 P2]*
Parents should be able to choose which conditions to include	*Quote 5: “I’m all about choice, especially in healthcare, but informed choice and decision making…” [FG7 P2]*
Conditions should be ‘grouped’ to allow for easier decisions	*Quote 6: “I think categories [of conditions] would be great, but then that has to come along with extra education, extra counselling in the beginning so that people can make a choice. So yes, I think it’s a good idea, but it’s another level of informing the public about what they can choose, what they can opt out of, what the consequences are if they do opt out of certain things.” [FG3 P1]* *Quote 7: “…maybe it needs to be a two-part testing where you can opt to have just the first bit where, you know, it’s curative or you’re preventing, you know, severe disease and disability, or you’re all in.” [FG7 P2]*

### Criteria for which conditions to include

Focus groups discussed different criteria for deciding which conditions to include in gNBS, such as age of onset, likelihood of the condition developing (i.e., penetrance), severity, treatability, variability, frequency, and accuracy of the test for each condition. Illustrative quotes are shown in Table 3.

Some participants felt that only conditions that had a treatment or intervention available should be included in gNBS (Table 3, Quote 1). These participants suggested that knowing about untreatable conditions would have significant psychological impact on families and even children themselves. However, many participants saw benefit in also including conditions for which no treatment was available (Table 3, Quote 2). They expressed that even if a condition was untreatable, there may be intervention, management, and peer support available. They also recognised that just because there was no treatment currently available, there may be in the future.

In relation to adult-onset conditions in particular, participants displayed strong views in both directions about whether or not they should be included, with some feeling that adult-onset conditions were not appropriate to screen at the newborn stage (Table 3, Quote 3), and others seeing high value in knowing this information early in life (Table 3, Quote 4). Irrespective of their preferences, participants agreed that if prioritisation of conditions was necessary, childhood-onset conditions were more important to include than later-onset conditions.

When prompted to discuss whether penetrance of a condition (the likelihood of it developing) was an important factor in deciding whether it should be included in gNBS, views again were mixed. While some stated that all conditions, irrespective of the likelihood of the condition developing, were important to include (Table 3, Quote 5), others conveyed a strong preference for prioritising conditions with a higher certainty of developing (Table 3, Quote 6). Participants also recognised the increased parental anxiety associated with receiving such an uncertain result from gNBS, and that estimates of penetrance may not be accurate or even available for all conditions.

While participants recognised that the severity of a condition may not always be able to be accurately predicted, some viewed this as an important factor in deciding whether to screen for the condition at birth (Table 3, Quotes 7 and 8). Others preferenced all conditions equally, regardless of their severity (Table 3, Quote 9). Participants had trouble deciding whether to include conditions for which severity was hard to predict (Table 3, Quote 10).

Participants were divided on whether the frequency of a condition should determine whether it is included in gNBS. Some felt that more common conditions should be prioritised, whereas others recognised the importance of including rare and very rare conditions as well (Table 3, Quote 11).

Participants discussed their concerns regarding the accuracy of gNBS, with several suggesting that only conditions for which an accurate genomic result could be produced should be included (Table 3, Quote 12). They expressed concern for potential unnecessary anxiety and distrust that low levels of test accuracy would generate. However, others were more open to receiving false positives and false negatives if it meant receiving more information about conditions overall (Table 3, Quote 13).

In addition to certain criteria for deciding whether to include a condition in the gNBS panel, some participants suggested that screening could also be based on family history (Table 3, Quote 14). However, some saw issues with this in that both biological parents might not always be available to provide family history information, and for some conditions there may not be a prior family history. While there were many views about what should and should not be included, when asked to prioritise, participants generally supported childhood onset, severe, treatable, highly penetrant conditions being prioritised.

**Table 3:** Criteria for which conditions to include

Criteria	Illustrative quote
Treatability of the condition	*Quote 1: “We know that we can screen for many different diseases but we don’t have treatments and cures for many diseases so you’re potentially sort of coming up with a genetic diagnosis at a young age for a young child with the parents then being faced with not having any treatment for that child or for treatment outcomes I think would be my main hesitation I think with offering it as a newborn level.” [FG6 P4]* *Quote 2: “I think it’s important not to just identify cases where there is a treatment because I do still think it’s significant for families regardless of treatment…if you do have a child that has a terminal diagnoses it’s still important that odyssey to find out what it is.” [FG1 P1]*
Age of onset of the condition	*Quote 3: “I think that that’s something that adults should be allowed to decide for themselves…I don’t think it’s up to the parent to decide that it’s their right to obtain that information. That’s not something that I would want my parents to decide for myself at an early age…” [FG7 P2]* *Quote 4: “I think even if it’s something that’s going to affect someone later in life, it should be detected early in childhood because it could be a family history there that could be detected through this and it could make life a bit easier for the adults if it is detected.” [FG2 P1]*
Penetrance (likelihood of developing the condition)	*Quote 5: “I think if you’re telling people or families that their child may or may not be affected by a disorder…I think that if they are then told what the expected course of the disease might be then they may recognise the symptoms earlier than they would have if they didn’t know…I think it leads to a better life for that child if the parents know what to look out for than if you don’t tell them.” [FG1 P4]* *Quote 6: “…if I had a child and I was told, “You know, they may have this really terrible incurable condition, but they may not,” I, as a person with anxiety, would be stressing about that 24/7. That is the type of thing that keeps me up at night, so I would stress a lot and I can imagine there would be other people like me out in the world that would be just worried all the time about it.” [FG10 P1]*
Severity of the condition	*Quote 7: “…it should be based on the seriousness of the condition that’s being tested for…” [FG5 P3]* *Quote 8: “And for me, I would want to know, the most important ones would be the more debilitating conditions, anything that’s going to affect my child’s mortality, and anything that’s going to affect their ability to have, again, normal-ish life.” [FG5 P6]* *Quote 9: “Just because something is considered mild, doesn’t mean it may or may not need support in the future…And so I think whether they’re mild or more severe cases of each thing, it would still be important to be able to get support for these kinds of things. I suppose just in the future for that child because mild things in anything really doesn’t mean you don’t need help or support with that.” [FG12 P5]*
Variability of the condition	*Quote 10: “But also there’s a lot of conditions that have a lot of variability…Where some people will go through their whole lives and not know they even have it, but some people just get unlucky.” [FG8 P4]*
Frequency of the condition	*Quote 11: “…there’s so many conditions out there…I guess maybe I could say, yeah, the ones that there’s lots of them, but then again maybe it’s more important to recognise the ones that there’s not lots of them…” [FG15 P3]*
Accuracy of gNBS for the condition	*Quote 12: “…unless it’s completely accurate and we know what’s really likely to happen, it’s that, the alarm factor and who wants to be unnecessarily alarmed or feel unnecessarily alarmed if the accuracy of the initial testing is not as great as it should be. Is genomics ready enough to provide such definite answers now?” [FG9 P1]* *Quote 13: “I think in every experiment, there are always chances of those undesired results. So the more we can reduce the probability of having those false negative or false positive, it’s better. And I think with time, it will improve, in my opinion.” [FG13 P1]*
Testing based on family history	*Quote 14: “I’m more thinking from the funding point…maybe there’s an option like Medicare in future, maybe can fund some of the general conditions testing for everyone, then the parents can base it on family medical history to select certain types to add onto these, applicable to everyone, like they have option to have a general test and plus a specific test for their own family conditions.” [FG10 P2]*

### Cost and funding

Focus groups discussed their views on how gNBS could be funded. Illustrative quotes are shown in Table 4.

Overwhelmingly, participants agreed that gNBS should be fully publicly funded, and that parents should not be asked to pay anything out of pocket to access screening (Table 4, Quote 1). This appeared to be motivated by the impression that if parents were asked to pay, significant health inequities could arise as a result. In fact, participants perceived that gNBS had potential to save the health system money in the long run and was therefore worthy of government funding (Table 4, Quote 2). They recognised that early intervention reduced longer term healthcare costs, and so felt gNBS would be beneficial in this regard.

Alternative models of funding suggested by participants included partially subsidising gNBS through government funding (Table 4, Quote 3). Several different approaches were suggested, including partially subsidising the screening test for all children; means-testing screening (i.e., subsidising only for disadvantaged families); subsidised for certain groups such as those with a family history (Table 4, Quote 4); or subsidising only a small set of conditions (Table 4, Quote 5), and allowing families to pay to include additional conditions.

Participants suggested that gNBS is so important that until public funding is available, as an interim measure, parents should have the option of gNBS even if it means they have to pay (Table 4, Quote 6).

Participants raised that if the government were to fund gNBS, funding would also need to cover support for babies diagnosed with genetic conditions through gNBS (Table 4, Quotes 7 and 8). These included treatments and therapies for the conditions diagnosed through gNBS, which would add to the overall cost of the program.

**Table 4:** Cost and funding

	Illustrative quote
gNBS should be publicly funded	*Quote 1: “I don’t think you could ethically offer something like this and not make it accessible to all, or just make it accessible for those who could afford it. It would be a non-starter. I would rather not have that system at all, if it’s only going to be available to people who had middle-class income or higher.” [FG5 P1]* *Quote 2: “…not just the intervention and further costs down the track, but also the diagnosis, that can be very costly because there’s a range of specialists and people involved to determine what the issue could be. So if that was determined from birth, that would be a great cost benefit.” [FG11 P1]*
gNBS could be partially subsidised	*Quote 3: “I wonder as well if there’s any option, like…if you are financial disadvantaged, could Medicare cover this for you? And then if you have the money to pay for it you just have to pay for it yourself if you want it.” [FG2 P4]* *Quote 4: “…if there has to be criteria around it, then it should be put back into I guess that documented medical history of family, or immediate family around it.” [FG5 P2]* *Quote 5: “Obviously it would be nice if it was all covered by Medicare…But in our realistic world, it sounds like it’s going to be super-duper expensive. So yeah, I think a compromise to that would be, “Okay, here are the, we’re going to test for these top 50 conditions, and then if you want to test for this and this and this, it’s optional, but it’s an extra charge,” and people can decide for themselves.” [FG5 P6]*
gNBS should still be offered even if people have to pay	*Quote 6: “…if it could be available earlier on to some people, like do you withhold it from some people who can pay, just because others can’t? I think that’d be, you’ve got, there’s a whole lot of moral dilemmas there.” [FG3 P2]*
Funding is required for support following a diagnosis from gNBS	*Quote 7: “…having the information is all very well, but you need the services available to actually offer the therapy and so on.” [FG8 P1]* *Quote 8: “…potentially there’s going to be more families that get a diagnosis early on in a fairly tricky, a fairly challenging time of parenting…And just ensuring that there’s the workforce to support that I think would be really important.” [FG15 P2]*

## Discussion

This work highlights important tensions that our participants held about potential trade-offs between cost and which conditions to offer as part of a gNBS program. A strength of our approach was that we did not limit discussions by proposing specific ways to decide which conditions should be screened or how to fund gNBS programs. This enabled participants to propose their own suggestions and revealed some of their underlying assumptions about the necessity to limit the number of conditions to be screened.

### Could parents be allowed to choose which conditions to include in gNBS?

For the participants in our study, decisions about which conditions to include in a gNBS program rested on whether parents would be able to make choices about which conditions were screened in their child within the program. Regarding parental choice about conditions included in the gNBS program, participants identified two potential models: (1) the program screens for a set list of conditions (i.e., no parental choice); and (2) the program allows some flexibility for individual parents/families to decide which conditions are screened. In the former option, participants assumed that some external expert(s) would decide, whereas in the latter, it is unclear whether participants appreciated that although each parent or family would be allowed choice, this would still be from a set, albeit longer (and with less stringent criteria) list of expert-approved conditions.

Allowing parents to choose which conditions they want to screen their infant for would impact service delivery in several ways. Allowing such individualisation of screening panels will require greater education of parents and greater support for their decision making. It would impact the level of informed consent required for gNBS, potentially requiring a more detailed consent process the more conditions are offered, affecting scalability and creating complexity in testing processes. Our participants suggested ‘grouping’ conditions to allow such decisions to be made more easily by parents. This aligns with the proposals of others of a tiered approach to informed consent for genomic sequencing, whereby individuals can opt to receive results from some tiers (or groups of conditions) and not others [12]. 

### If the set list of conditions is limited, which ones should be included?

If instead screening is limited to a set list of conditions, which parents could either choose to opt-in or opt-out of as a whole, the decision about which conditions should be screened is perhaps even more critical. In our study, participants saw benefits in screening for all types of conditions, including those which were untreatable, mild, variable, and had onset in adulthood. However, if asked to prioritise, participants recognised that treatable, severe, early-onset, and highly penetrant conditions should be included over others.

In deciding which conditions to include in this type of list, lessons can be learned from other population-wide genomic sequencing programs such as reproductive carrier screening [13] and other gNBS projects [14–18]. Gene list development for such other programs has involved consultation with a variety of stakeholders, including clinical geneticists, genetic counsellors, laboratory geneticists, and other specialists [13–18]. Some have engaged ethicists and parents [13] as well as the public [14] in the decision-making process, although patient support organisations should also be included in these discussions.

Existing gene lists proposed for gNBS programs are highly variable, but tend to be based on similar criteria of test validity, treatability, and age of onset of disease [15–18]. These programs have generally chosen to include only conditions which are severe (having a significant risk for morbidity or mortality [15, 18]), have onset in early childhood [15–18], and for which an effective treatment or intervention is available [15–18]. Recommendations from bodies such as the Global Alliance for Genomics and Health also suggest that gNBS should be limited to conditions with early onset and that would benefit from early intervention [19]. 

Other gNBS programs have also recognised the importance of having accurate testing for all conditions or variants screened, with strong supporting evidence for genotype-phenotype correlation [15, 16, 18]. Our focus group participants were cognisant of the potential increased anxiety that receiving an uncertain or inaccurate result could cause new parents, and wanted reliable results for conditions if they were to be included. This may reflect the public’s, at time, unrealistic expectations of the accuracy of genomic sequencing in this setting and therefore the readiness of inclusion of genomics in newborn screening. Although research is needed to assess these misconceptions in more detail, this provides us with some insight into the future educational needs prior to implementation. Interestingly, although one study suggested false positive results for standard NBS were associated with emotional distress for parents [20], others have shown no difference in anxiety or stress in mothers receiving false positive results compared with true positive or negative results [21]. This discrepancy is likely to be because the distress was primarily due to poor communication of the information by the healthcare provider [22]. Finally, frequency of a condition was not a strong deciding factor for participants as to whether it should be included in gNBS panels; this is reflected in other gNBS programs, whereby conditions are not prioritised based on how common or rare they are [15–18]. 

#### Including untreatable conditions

Interestingly, participants’ desire to include both treatable and untreatable conditions in a gNBS panel does not align with current recommendations, nor most existing gNBS programs (with the BabySeq Project in the US an outlier [16]). The question of whether to include untreatable conditions in such programs is contentious as guidelines state that the primary purpose of NBS should be early intervention (and even prevention) for conditions with treatability as a key criteria [19]. Yet results previously published from this study show that participants recognise that knowing about conditions for which no treatment is available also has benefits, such as: the avoidance of a lengthy diagnostic odyssey and unnecessary testing and investigations; allowing time for parents to adjust to the diagnosis and make changes to their lifestyle accordingly; and providing information which may be used for reproductive planning [8]. This raises questions about whether it is justifiable not to offer to screen for untreatable conditions when some parents would find it valuable and it is possible to do so. It also highlights the challenges of determining the extent to which public perspectives can and should be incorporated into screening programs and/or policy. Further work is needed to assess whether prospective parents would actually choose to receive information about untreatable conditions if they were available in order to help determine whether it is worth exploring offering it.

Even if there is agreement that treatability is a critical criterion, determining what this means is not straightforward. Others have examined the concept of treatability (or actionability) in the context of genomic secondary findings [23]. Richer and colleagues suggest that the definition of actionability in childhood should be based on the proportion of cases that present in childhood, as well as the quality of the evidence supporting any available treatments or intervention [23]. Interestingly, our participants saw that treatability could mean anything from a medication available to an early intervention measure (such as additional education support). These various concepts of treatability or actionability further complicate decisions about criteria for deciding which conditions to include in gNBS. This was indeed the most common reason for discrepancies between existing gNBS panels in a recent comparison [15], and therefore a factor that will need to be considered carefully based on both current research and local context.

#### Assessing severity

While our participants saw benefits in screening for less severe conditions, most gNBS programs prioritise severe conditions over their mild counterparts [14, 15, 18, 19]. However, some authors have raised concerns over the very concept of severity, noting that a clear definition is lacking [24]. Newson and Dive suggest that using a concept such as severity (or seriousness) of a condition requires deep ethical and conceptual analysis and should take into account the views of health professionals and those living with the condition [24]. Importantly, they highlight that conditions should not be categorised into universally-applicable ‘buckets’ based on severity, but that descriptions of severity instead need to incorporate complex social, cultural and environmental factors unique to each context and potentially each individual impacted.

These varying perceptions of severity may be reflected in our data, where participants had diverse views about whether severity should be used as a factor in deciding whether to include a condition in a gNBS panel. Furthermore, participants’ perceptions of severe appeared to include concepts ranging from ‘debilitating’, ‘affecting mortality’, or ‘affecting a child’s ability to have a normal life’ [FG5 P6], to simply ‘needing support’ [FG12 P5].

### Cost and funding

Ultimately, decisions about which conditions to include in gNBS panels – and how much flexibility to allow individual families to pick and choose the results they want to receive – may come down to funding. Traditionally, in public health programs, funding is limited and prioritisation of what is offered is required [19]. Programs with greater in-built individualisation are likely to require greater resources to implement and deliver both the service and its downstream consequences. A trade-off may therefore be required between tailorability and population-wide benefit.

In Australia, standard newborn screening (stdNBS) is funded for all infants [25]. However, its costs are low, at less than $5 per infant, including downstream costs [26]. gNBS is likely to be at least 50 times more expensive [5]. Despite knowing this, in focus groups, participants demonstrated strong preferences for gNBS to be publicly funded to mitigate potential inequities and to benefit the taxpayer overall by saving healthcare costs in the long term. They also wanted additional public funding to be allocated for support services for those who receive a diagnosis of a genetic condition through gNBS. Other guiding bodies have highlighted justice and equity of access as a key consideration in the development of gNBS programs, reflecting that the success of such programs is predicated on their availability to all [19]. Similarly, Genomics England identified equity of access to treatment as a key principle guiding decisions about which conditions to include in their gNBS panel; that is, they concluded that conditions screened for should only include those for which the interventions are equitably accessible for all [14]. 

Some parallels may be drawn here between gNBS and the introduction of other new genomic technologies, such as prenatal cell-free DNA (cfDNA) screening and reproductive genetic carrier screening. In Australia, screening for chromosomal conditions during pregnancy is subsidised by the government for all pregnant people in the form of first-trimester combined screening [27]. Prenatal cfDNA screening – a more accurate screening test available slightly earlier in pregnancy – is available at a cost to families, with no government subsidisation currently available [27]. Similarly, various forms of reproductive genetic carrier screening – from only a few to hundreds or thousands of conditions – is available at a cost to prospective parents in Australia, while screening for the three commonest conditions (cystic fibrosis, spinal muscular atrophy and Fragile X syndrome), is publicly funded [28]. 

Despite their strong desire for public funding, participants recognised that partial government subsidisation or private payment may be required as an interim measure. Other gNBS programs have approached this issue by using gNBS as an adjunct to stdNBS, rather than a replacement, as supported by global recommendations [19]. While most stdNBS programs screen for less than 50 conditions, genomic sequencing technologies allow the inclusion of hundreds, if not thousands, of additional conditions, at potentially very little incremental cost. Deciding which – and how many more – conditions to offer for screening presents a fine balance between cost, ethics, and practical limitations of testing. We may consider that, at least at the outset, gNBS could follow a similar implementation trajectory to other new genomic technologies – such as prenatal cfDNA screening and reproductive carrier screening – whereby stdNBS is still funded for all infants, and gNBS is offered as an additional option to those willing to pay. However, this is likely not ethically appropriate in the long-term; should gNBS be shown to improve health outcomes, all babies should have access to this technology.

## Conclusion

The increasing availability and decreasing cost of genomic sequencing technologies has led to many discussions about expanding the number of conditions screened for in NBS programs. Our findings show that members of the public generally support expansion of the number of conditions that are included in newborn screening programs and inclusion of both treatable and untreatable childhood onset conditions. While it is tempting to adopt a more inclusive, broader approach to such screening programs, we must consider the potential impact that offering a wider scope of conditions may have on uptake of screening. It is therefore important that decisions about which conditions to include carefully consider both expert and public opinion. Our study provides insight into these opinions of the Australian public in order to inform such decisions.

How many conditions are included in a gNBS program will be a complex decision requiring detailed evaluation of costs and benefits alongside public and professional engagement. Our participants were highly in favour of gNBS being government funded, although they did appreciate the challenges associated with this. Future work should examine the nuances of the trade-offs required between cost, ethics, and practical limitations of screening to provide a gNBS program that meets the expectations of all key stakeholders whilst maintaining the high public trust currently held in stdNBS.

### Electronic supplementary material

Below is the link to the electronic supplementary material.

Supplementary Material 1

## Data Availability

The datasets used and/or analysed during the current study are available from the corresponding author on reasonable request.
