# Supplementary material for: Australian public perspectives on genomic newborn screening: which conditions should be included?
Source: Hum Genomics. 2024 May 8;18:45. doi: 10.1186/s40246-024-00611-x (PMC11077791; doi:10.1186/s40246-024-00611-x)
Supplement: Supplementary file 1 — Supplementary Material 1 [file 40246_2024_611_MOESM1_ESM.docx]

**Focus group guide**

**How should GNBS be implemented?**

**Welcome**

**Introductions**

**Purpose of this Session**

**Housekeeping**

**Brief Refresher on Material from the Video**

**Group Discussion Part 1** – Introductions and warm up questions

**Polls**

1. Do you think genomics should be used in newborn screening programs?

Yes No Not sure

1. Should a newborn screening program that includes genomics be run any differently to standard newborn screening programs?

Yes No Not sure

**Group Discussion Part 2**

1. Why did you answer yes on no to whether genomics should be used in newborn screening programs?

2. If you answered no to running newborn screening programs that include genomics differently, why?

3. If you answered yes to running newborn screening programs that include genomics differently, what do you think should be done differently?

Now, let’s talk about how parents (or prospective parents) should be told about genomic newborn screening.

4. When should genomic newborn screening be initially discussed with parents?

5. Who should tell the parents about genomic newborn screening?

6. Where should genomic newborn screening be discussed with parents?

Let’s talk about the way we should ask permission for genomic newborn screening to take place. This is also known as giving consent.

7. Do you think parents need to give consent for genomic newborn screening or should testing happen automatically? Why?

8. Should the consent process be any different from standard newborn screening? Why/why not?

9. When do you think parents should be asked to give consent for genomic newborn screening to take place for their baby?

10. What information do you think is important to know to help parents make decisions about genomic newborn screening for their baby?

11. How should information be provided?

Now let’s talk about some of the factors we might want to think about when deciding which genetic conditions to screen for.

12. What features of conditions do you think are important when making decisions about what to include?

13. What if you could know about many of these conditions before you got pregnant?

Which types of conditions would you prefer to know about beforehand?

What would you do with that information?

14. Who should pay for the genomic newborn screening?

**Group Discussion Part 3 – Assessing risks and benefits**

1. What are your thoughts on whether genomics should be used in newborn screening?
2. What do you see as some of the advantages of using genomics in newborn screening?
3. What do you see as some of the potential risks of using genomics in newborn screening?
4. What do you think is important when thinking about using genomics in newborn screening?
5. Do you think genomic data should be stored after it has been used in screening?
6. Is there anything else we have missed?

Polls

1. Do you think genomics should be used in newborn screening programs?

Yes No Not sure

2. Should a newborn screening program that includes genomics be run any differently to standard newborn screening programs?

Yes No Not sure

**Wrap up**

- Final comments and thoughts from participants
- Summary of discussions
- Next steps
